# Patients Use Fewer Opioids Than Prescribed After Arthroscopic Release of Elbow Contracture: An Evidence-Based Opioid Prescribing Guideline to Reduce Excess

**DOI:** 10.1016/j.asmr.2021.09.002

**Published:** 2021-11-17

**Authors:** Jorge Rojas Lievano, Dani Rotman, Maegan N. Shields, Mark E. Morrey, Joaquin Sanchez-Sotelo, Dave R. Shukla, Tammy S. Olson, Anthony M. Vaichinger, James S. Fitzsimmons, Shawn W. O’Driscoll

**Affiliations:** Department of Orthopedic Surgery, Mayo Clinic, Rochester, Minnesota, U.S.A.

## Abstract

**Purpose:**

To generate an evidence-based opioid-prescribing guideline by assessing the pattern of total opioid consumption and the factors that may predict opioid consumption following arthroscopic release of elbow contracture and to investigate whether the use of continuous passive motion (CPM), as compared to physical therapy (PT), was associated with a decrease in pain and opioid consumption after arthroscopic release of elbow contracture.

**Methods:**

Data collected from a randomized controlled trial that compared continuous passive motion (CPM) (*n* = 24) to physical therapy (PT) (*n* = 27) following arthroscopic release of elbow contracture was analyzed for opioid use. Fifty-one participants recorded their daily opioid consumption in a postoperative diary for 90 days. Multivariate analysis was performed to identify factors associated with opioid use. Recommended quantities for postoperative prescription were generated using the 50th percentile for patients without and the 75th percentile for patients with factors associated with higher opioid use.

**Results:**

The median total opioid prescription was 437.5-mg morphine milligram equivalents (MMEs) (58 pills of 5 mg oxycodone) and the median total opioid consumption was 75 MMEs (10 pills of 5-mg oxycodone). Twenty-two percent of patients took no opioid medication, 53% took ≤10 pills, 69% took ≤20 pills and 75% took ≤30 pills. Predictors of higher opioid use were preoperative opioid use, age <60 years and inflammatory arthritis. The total opioid consumption appeared similar between the CPM and the PT group. Seventy-five percent of patient’s home opioid requirements would be satisfied using the following guideline: Patients undergoing contracture release for osteoarthritis or post-traumatic contracture should be given a prescription for 10 pills of 5 mg oxycodone or its equivalent at discharge. Patients with inflammatory conditions or those taking preoperative opioids should be prescribed 30 pills of 5 mg oxycodone or its equivalent.

**Conclusion:**

This study suggests that most patients undergoing arthroscopic release of elbow contracture use relatively few opioid pills after surgery. Use of an evidence-based guideline could decrease opioid prescriptions substantially, while still effectively treating patients’ pain.

## Introduction

Since 2012, the overall opioid prescribing rate in the United States has been declining as a result of multiple efforts to increase awareness among providers and patients of the risks associated with opioid use.^1^ However, despite reductions, currently, the amount of opioids prescribed remains approximately three times higher than in 1999.^1^ As managers of acute postsurgical pain, orthopedic surgeons can play an important role in further decreasing the quantity of prescribed opioids in the United States.^2^

For many years open capsular release had been the standard treatment for elbow contractures.^3^ However, an increasing trend in arthroscopic elbow procedures has been observed in the United States over the last several years,^4^ and the use of arthroscopy for elbow contracture release has become more popular.^3^^,^^5^, ^6^, ^7^, ^8^ Despite the increase in the use of arthroscopic elbow procedures, there is a paucity of data to inform appropriate opioid prescribing for patients who undergo common arthroscopic elbow procedures, such as contracture release.

The purposes of this study were to generate an evidence-based opioid-prescribing guideline by assessing the pattern of total opioid consumption and the factors that may predict opioid consumption following arthroscopic release of elbow contracture and to investigate whether the use of continuous passive motion (CPM), as compared to physical therapy (PT), was associated with a decrease in pain and opioid consumption after arthroscopic release of elbow contracture. We hypothesized that the majority of patients would require far fewer opioid pills than were prescribed to them and that patients who received CPM would consume significantly fewer opioid pills than patients who received PT.

## Methods

This study was a secondary analysis of data prospectively collected as part of a randomized controlled trial (RCT) at the Mayo Clinic (Rochester, MN) comparing the effectiveness of CPM to PT for rehabilitation following arthroscopic release of elbow contractures. Opioid consumption and pain were exploratory outcomes of the RCT, and, as such, they were analyzed in a post hoc fashion.

Following institutional review board approval, this RCT was conducted at a single academic medical center from December 2016 through April 2019. Patients who were 13 years of age or older were eligible for inclusion if they had a lack of elbow flexion and/or extension, causing functional impairment that had been present for at least 6 months, had failed to respond to nonsurgical treatment, and were surgically treated with an arthroscopic capsulectomy or osteocapsular arthroplasty. Three surgeons participated in the study, though all surgeries were performed by one surgeon. Patients with contraindication to use of CPM or regional brachial plexus block, progressive or recurrent contracture due to inflammatory disease or chondrolysis, progressive or recalcitrant neuropathy, and those with altered anatomy that might limit elbow motion independent of the condition being treated were excluded. A complete list of exclusion criteria is provided in the Supplemental File.

**Fig 1:**
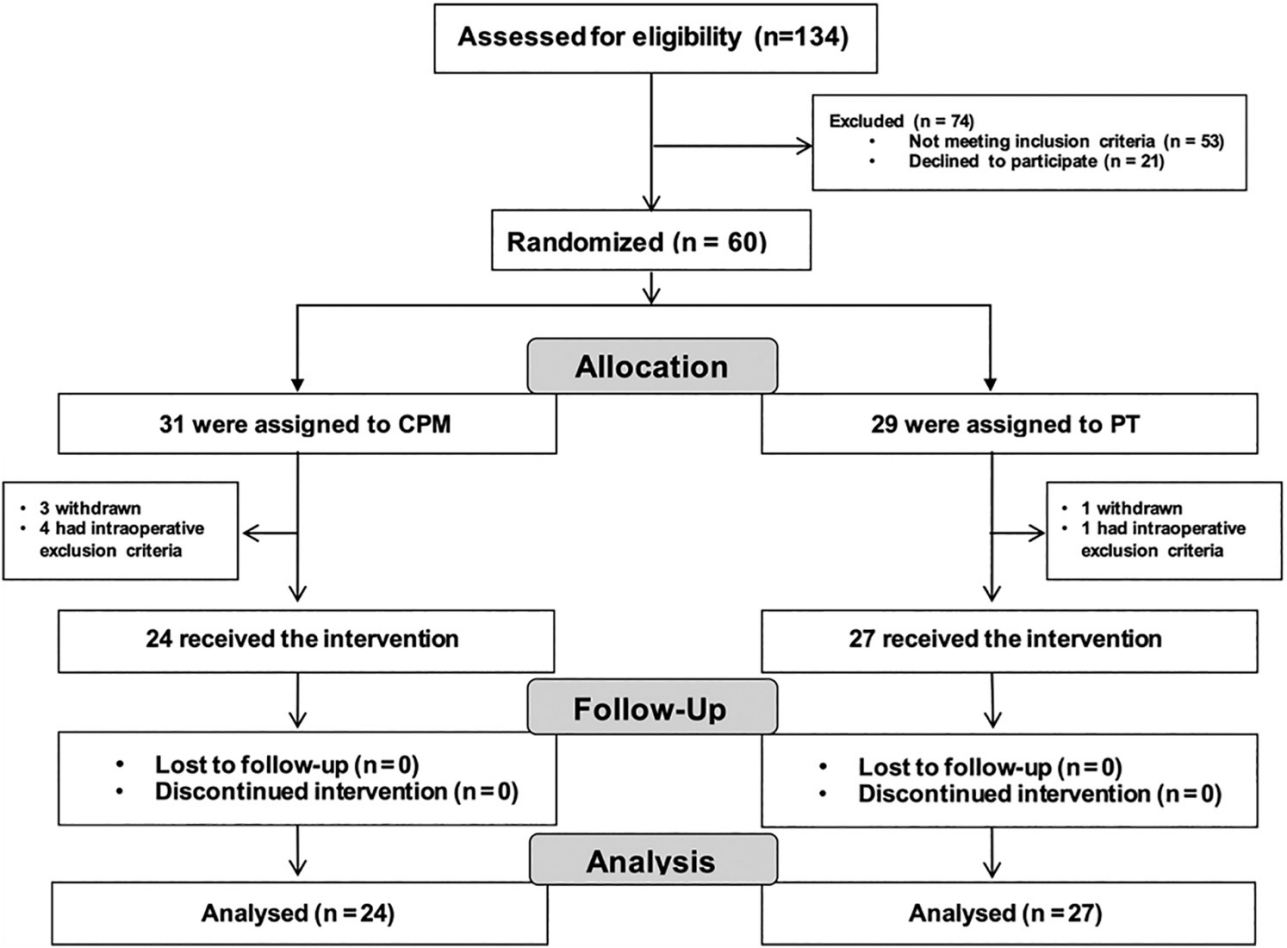
Flow diagram for enrollment, randomization, and follow-up.

Of the 134 patients who were screened, 60 underwent randomization; 31 were assigned to the CPM group, and 29 were assigned to the PT group (Fig. 1). Four patients withdrew consent to participate in the study, and 5 had intraoperative exclusion criteria, which left 24 patients in the CPM group and 27 in the PT group. At baseline, demographic data, preoperative pain severity (ASES-elbow pain score), severity of elbow contracture, smoking status, preoperative opioid usage, presence of ulnar neuropathy symptoms, subjective functional status (ASES-elbow function score), and subjective anxiety/depression status (EQ-5 anxiety/depression) were collected. Baseline characteristics were similar in the two groups (Table 1).

**Table 1:** Demographic and Clinical Characteristics of Patients at Baseline∗

Characteristic	CPM Group (*n* = 24)	PT Group (*n* = 27)	All Patients (*n* = 51)
Age, years			
Mean	50 ± 11	47 ± 18	48 ± 15
Range	13-65	14-71	13-71
Sex – no. (%)			
Male	21 (87)	22 (81)	43 (84)
Female	3 (13)	5 (19)	8 (16)
Elbow contracture etiology – no. (%)			
Primary osteoarthritis	16 (67)	13 (48)	29 (57)
Post-traumatic	6 (25)	10 (37)	16 (31)
Inflammatory	2 (8)	4 (15)	6 (12)
Preoperative arc of elbow motion-degrees			
Mean	83 ± 26	80 ± 16	82 ± 21
Range	5-110	50-115	5-115
Severity of elbow contracture – no. (%)†			
Mild (arc > 90°)	10 (42)	7 (26)	17 (33)
Moderate (arc 61°–90°)	11 (46)	18 (67)	29 (57)
Severe (arc 31°–60°)	1 (4)	2 (7)	3 (6)
Very severe (arc ≤ 30°)	2 (8)	0 (0)	2 (4)
History of previous surgery for elbow contracture – no. (%)			
No	21 (87)	21 (78)	42 (82)
Yes	3 (13)	6 (22)	9 (18)
Score on the numeric rate score (NRS) for pain at worst			
Mean	6.1 ± 3.3	7.2 ± 2.3	6.7 ± 3
Range	0-10	0-10	0-10
Ulnar nerve neuropathy – no. (%)			
No	17 (71)	16 (59)	33 (65)
Yes	7 (29)	11 (41)	18 (35)
Preoperative opioid usage – no. (%)			
No	21 (88)	26 (96)	47 (92)
Yes	3 (12)	1 (4)	4 (8)
Smoking status – no. (%)			
Nonsmoker	22 (92)	25 (93)	47 (92)
Current smoker	2 (8)	2 (7)	4 (8)
Total score on the ASES-elbow function			
Mean	26 ± 8	25 ± 7	25 ± 7
Range	7-36	8-36	7-36
Total score on the ASES-elbow pain			
Mean	23 ± 12	28 ± 11	26 ± 12
Range	2-44	1-46	1-46
EQ-5D Anxiety/Depression status – no./total no. (%)‡			
No problems	18/24 (75)	24/26 (92)	42/50 (84)
Any problem	6/24 (25)	2/26 (8)	8/50 (16)

ASES, American Shoulder and Elbow Surgeons; EQ-5D, EuroQol five-dimensional.

∗ Plus–minus values are means ± SD. The recruited patients were randomly assigned to receive continuous passive motion (CPM) or physical therapy (PT) as rehabilitation protocol after arthroscopic OCA. There were no significant between-group differences in the demographic and clinical characteristics of the patients at baseline.

† Severity of elbow contracture was determined according to previous publication on release of elbow contractures.

‡ Data on the EQ-5D anxiety/depression domain at baseline was missing for one patient in the PT group.

Arthroscopic contracture release was performed by a single surgeon in a standard fashion following a surgical technique that has been previously described in detail.^8^^,^^9^ The ulnar nerve was addressed in all but three patients who had previously undergone ulnar nerve transposition and did not have any ulnar nerve symptoms at the initial assessment. In 41 patients who did not have ulnar neuropathy symptoms at the initial assessment, a limited decompression of the ulnar nerve was performed at the time of release in order to prevent delayed onset ulnar neuritis.^10^ Subcutaneous ulnar nerve transposition was performed in seven patients who had ulnar neuropathy symptoms at the initial assessment. The transposition was performed in a staged manner to decrease the risk of wound-healing problems and hematoma formation^10^ in six of the seven patients (average time between transposition and release 15 weeks [range, 9–20 weeks]). These patients underwent ultrasound imaging of the transposed nerve the day before contracture release surgery in order to plan the proximal anteromedial portal. In one patient, the transposition was performed at the same time of contracture release due to patient’s preference for a single procedure. Ulnar nerve management, tourniquet time, and other surgical variables in the two groups are presented in Table 2.

**Table 2:** Surgical Characteristics∗

Characteristic	CPM Group (*n* = 24)	PT Group (*n* = 27)	All Patients (*n* = 51)
Type of elbow contracture release – no. (%)			
Osteocapsular arthroplasty	23 (96)	23(85)	46 (90)
Capsular release (soft tissue only)	1 (4)	4 (15)	5 (10)
Ulnar nerve management – no./total no. (%)†			
Limited decompression	20/22 (91)	21/26 (81)	41/48 (85)
Subcutaneous transposition	2/22 (9)	5/26 (19)	7/48 (15)
Additional surgical procedures – no. (%)‡			
Removal of heterotopic ossification	4 (17)	3 (11)	7 (14)
Radial head excision with/without interposition arthroplasty	0 (0)	3 (11)	3 (6)
Hardware removal	0 (0)	2 (7.4)	2 (4)
Other procedures ∗∗	1 (4)	1 (4)	2 (4)
Tourniquet time – minutes			
Mean	91 ± 29	90 ± 26	90 ± 27
Range	33-129	49-140	33-140

∗ Plus–minus values are means ± SD. There were no significant between-group differences in the surgical characteristics.

† The ulnar nerve was not addressed in two patients in the CPM group and one patient in the PT group because they had previously undergone ulnar nerve transposition and did not have ulnar neuropathy symptoms at the initial assessment.

‡ Other procedures included recontouring distal humeral osteotomy in one patient in the PT group and open removal of medial forearm cyst with arthroscopic curettage of cyst in the capitellum in one patient in the CPM group.

After surgery, patients in the CPM group were admitted and received an indwelling axillary catheter for a continuous brachial plexus block for 48 hours. CPM was performed in the hospital for 3 days and at home for up to 4 weeks. While in hospital, patients in the CPM group received oral opioid medication if pain control was not achieved with the brachial plexus block. Patients in the PT group did not receive any regional anesthesia block and were discharged the same day. PT was performed daily for the first 3 days and then 3 times per week thereafter. A detailed description of the CPM and PT protocols is provided in the Supplemental File. Upon hospital discharge, patients received a prescription of indomethacin 25 mg 3 times daily for 21 days for heterotopic ossification prophylaxis, and an opioid medication for breakthrough pain. Additionally, patients were permitted to use any over-the-counter nonopioid analgesics as necessary. Opioid prescription included oxycodone 5-mg tablets and tramadol 50-mg tablets. Oxycodone was prescribed for a frequency of 1 to 2 tablets every 4 hours as needed. Tramadol was prescribed for a frequency of 1 tablet every 6 hours as needed. We did not have a standardized opioid prescription protocol for arthroscopic release of elbow contractures during the study period, and thus, the quantity of prescribed opioids was left to the discretion of the care team. The medical record was reviewed for the quantity of prescribed opioids, and the number and quantity of refills were provided within 90 days of surgery.

Starting from the first postoperative day and for 90 days postoperatively, patients completed a daily pain diary documenting their average pain level over the prior 24 hours on a numeric rating scale (NRS) scored from 0 to 10 points and pain medication consumption, both opioid and nonopioid analgesics (number of pills and dose). At the end of the 90 days, all 51 patients returned their completed diaries. To facilitate the analysis and interpretation, prescribed oxycodone and tramadol and their consumption were normalized into morphine milligram equivalents (MMEs) and an equivalent number of oxycodone 5-mg pills using conversion factors.

To generate an appropriate recommendation for discharge opioid prescription after arthroscopic release of elbow contracture, we arbitrarily decided to target the 75th percentile of the opioid consumption distribution for patients with risk factors associated with increased opioid consumption and the 50th percentile for patients without those factors.

### Statistical Analysis

Data were analyzed with descriptive statistics to determine the frequency for categorical variables and the mean, standard deviation, median, and quartiles for continuous variables, as appropriate for their distribution. A multivariate analysis was performed to identify the factors associated with increases in the number of pills taken postoperatively. Because of the number of opioid pills taken was nonnormal count data with a high proportion of zeros, a negative binomial model with robust standard errors was used for multivariate analyses. Variables with *P* < .25 in the univariate analysis were analyzed in hierarchical regressions, and model fit statistics were used to determine which variable to include in the final model. Variables from the hierarchical analysis with *P* < .05 were included in the final model. The difference in the median number of opioid pills consumed and the median time to opioid discontinuation between the CPM group and the PT group were tested using quantile regression. Statistical analyses were performed using Stata 14 (StataCorp. 2015, Stata Statistical Software: Release 14, College Station, TX, StataCorp LP) and JMP®, version 14.1.0 (SAS Institute Inc., Cary, NC).

## Results

### Opioid Prescribing and Consumption

**Fig 2:**
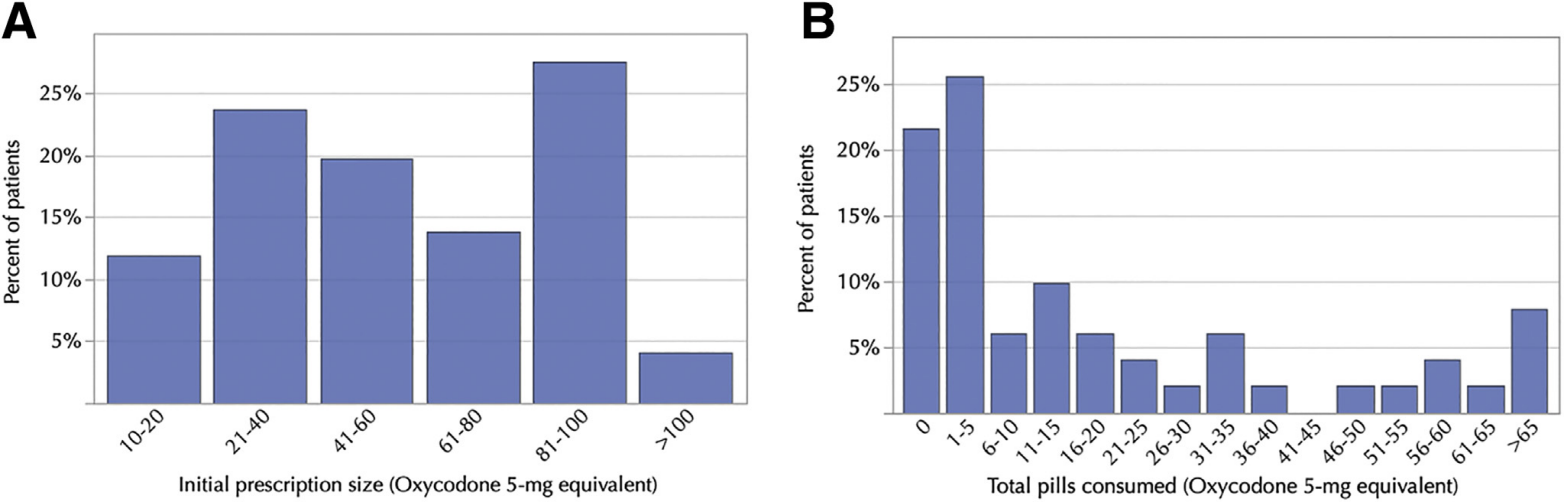
Distribution of initial prescription size (A) and total opioid consumption (B) (converted to oxycodone 5-mg pill equivalents).

The median total opioid prescribed was 437.5 MMEs (range: 75 to 1,000), which is equivalent to a median of 58 pills of 5-mg oxycodone (range: 10 to 133) (Table 3 and Fig 2A). The median total opioid consumption was 75 MMEs (range: 0 to 975), which is equal to a median of 10 pills of 5-mg oxycodone (range: 0 to 127) (Table 3 and Fig 2B). Twenty-two percent of patients took no opioid medication, 53% took ≤10 pills, 69% took ≤20 pills, and 75% took ≤30 pills. Fifty percent of patients had discontinued opioid usage by postoperative *day 4*, and 75% by postoperative *day 15*. Only one patient (2%) continued taking opioids at the end of the 90-day follow-up. Seventy-six percent of patients reported concomitant use of an over-the-counter analgesic. There was no association between use of an over-the-counter analgesic and opioid consumption (*P* = .41).

**Table 3:** Opioid Prescribing and Consumption Data

	CPM Group (*n* = 24)	PT Group (*n* = 27)	All Patients (*n* = 51)
Type of opioid prescribed– no. (%)			
Oxycodone 5 mg and tramadol 50 mg	16 (67)	13 (48)	29 (57)
Only oxycodone 5 mg	8 (33)	13 (48)	21 (41))
Only tramadol 50 mg	0 (0)	1 (4)	1 (2)
Number of pills prescribed∗			
Total	1520	1590	3110
Median	60	58	58
Range	10-117	13-133	10-133
Number of pills taken∗			
Total (%)†	465 (31)	602 (38)	1067 (34)
Median	8	13	10
Range	0-96	0-127	0-127
Prescription refill – no. (%)			
No	22 (92)	25 (93)	47 (91)
Yes	2 (8)	2 (7)	4 (9)

∗ Oxycodone 5 mg pill equivalents. There were no significant between-group differences in the median number of opioid pills prescribed or taken.

† Percentage of the initial quantity prescribed.

At the end of the 90-day follow-up, one-third (34%) of the opioid pills prescribed had been taken, and 96% of patients had opioids left over (median 40 pills, interquartile range 16-65). Seven patients (14%) completed their initial prescription, and 4 patients (8%) obtained an opioid prescription refill.

### Factors Associated With Higher Opioid Use

Results of univariate analysis are presented in Table 3. Multivariate analysis found that predictors of higher opioid use were diagnosis, age, and preoperative opioid usage. Patients who underwent surgery for inflammatory arthritis used 5 times more pills than patients who underwent surgery for osteoarthritis or posttraumatic contracture (95% confidence interval [CI], 1.7 to 13.3; *P* = .003). Patients <60 years old used 2 times as many pills as patients ≥60 years old (95% CI, 1.1 to 4.7; *P* = .03). Patients with a history of preoperative opioid usage used 3 times the pills of patients without preoperative opioid usage (95% CI 1.04 to 7.9; *P* = 0.04). No association was seen between the number of opioid pills used at home and sex, severity of contracture, smoking status, preoperative pain level, preoperative ulnar neuropathy, preoperative ASES-elbow function score, preoperative anxiety/depression status, history of previous surgery for elbow contracture, additional procedures at the time of contracture release, operative time, or rehabilitation protocol.

### CPM versus PT

**Fig 3:**
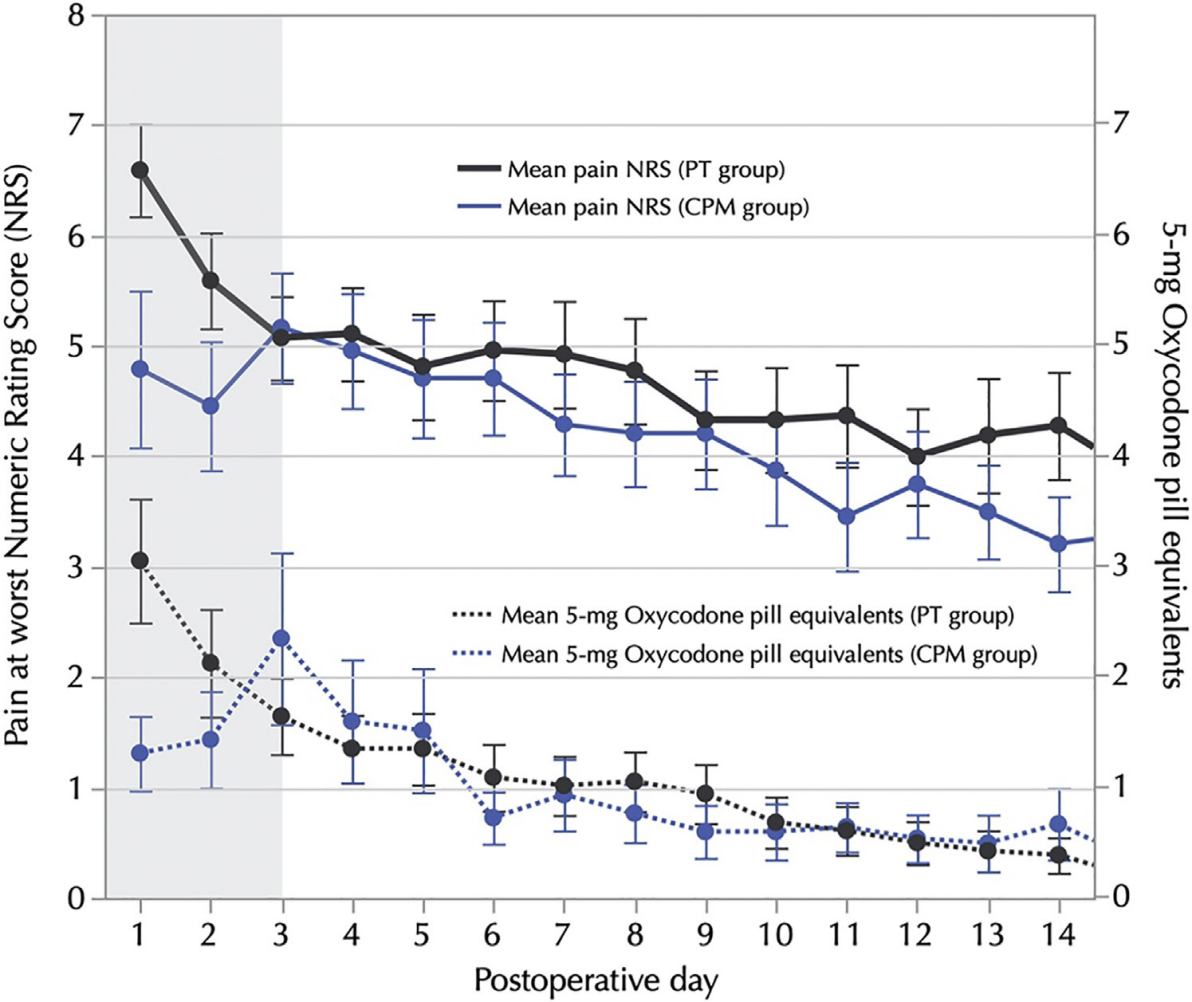
Mean numeric rating scores for pain at worst and mean opioid consumption (converted to oxycodone 5-mg pill equivalents) for each postoperative day within 14 days of surgery. Shadowed area represents the period of time patients in the CPM group were in hospital.

There was neither significant difference between groups in the total opioid consumption nor the time to opioid discontinuation. The median total opioid consumption in the CPM group was 60 MMEs (8 pills of 5 mg oxycodone) versus 92.5 MMEs (13 pills of 5 mg oxycodone) in the PT group (median between-group difference 32.5 MMEs; 95% confidence interval −71.1 to 136.1; *P* = .53) (Table 4). The median number of days to opioid discontinuation in the CPM group was 6 (range: 0-40) versus 4 (range: 0-90) in the PT group (median between-group difference 2 days; 95% confidence interval: −9 to 5; *P* = .59). The percentage of patients who took no opioids after surgery did not differ between groups (21% CPM group vs 22% PT group; *P* = .90). The daily opioid consumption and daily pain scores were lower in the CPM group than in the PT group during the first two postoperative days with no apparent differences between groups thereafter (Fig 3).

**Table 4:** Univariate and Multivariate Analysis of Variables Associated with Opioid Pills Taken Postoperatively

Variables	Univariate Analysis		Multivariate Analysis	
	IRR∗ (95% CI)	*P* Value	IRR∗ (95% CI)	*P* Value
Age				
<60 years	1.51 (.72-3.91)	.22	2.29 (1.12-4.67)	.03
≥60 years	Reference		Reference	
Sex				
Male	Reference			
Female	1.63 (.32-5.54)	.51		
Elbow contracture etiology				
Primary osteoarthritis	Reference		Reference	
Post-traumatic	.61 (.24-1.58)	.28	.72 (.31-1.69)	.39
Inflammatory	3.01 (1.53-6.41)	.002	4.96 (1.52-6.45)	.003
Severity of elbow contracture				
Mild (arc > 90°)	Reference		Reference	
Moderate and severe (arc ≤ 90°)	.92 (.41-1.98)	.86		
History of previous surgery for elbow contracture	1.38 (.58-3.12)	.38		
Preoperative ulnar nerve neuropathy	.82 (0.37-1.78)	.66		
Preoperative opioid usage	3.05 (1.41-6.88)	.004	3.01 (1.05-7.88)	.04
Smoking status				
Nonsmoker	Reference			
Current smoker	1.08 (.42-2.87)	.87		
Preoperative score on the ASES-elbow function	0.96 (.92-1.01)	.12		
Preoperative score on the ASES-elbow pain	1.03 (.99-1.06)	.08		
EQ-5D Anxiety/Depression status				
No problems	Reference			
Any problem	1.98 (.90-4.33)	.09		

ASES, American Shoulder and Elbow Surgeons, EQ-5D, EuroQol five-dimensional.

∗ The incidence rate ratio (IRR) represents the change in the dependent variable (number of opioid pills taken) in terms of percentage (determined by the amount the IRR is above or below 1) per unit increase of continuous independent variables, in the yes versus no for binary independent variables or in the category of interest versus category of reference for categorical independent variables.

### Recommendations for Discharge Opioid Prescription

Seventy-five percent of patient’s home opioid requirements would be satisfied using the following guideline: Patients undergoing arthroscopic release of elbow contracture for osteoarthritis or posttraumatic contracture should be given a prescription for 10 pills of 5 mg oxycodone or its equivalent at discharge. Patients undergoing OCA for inflammatory conditions or those taking preoperative opioids, should be prescribed 30 pills of 5 mg oxycodone or its equivalent. In this study, no significant differences were found between CPM and PT in terms of opioid consumption, and thus, the proposed guideline for opioid prescription should not vary based on the rehabilitation protocol used after surgery. One same-size refill can be given for each group, respectively, before considering other pain management strategies or referral to a pain services specialist, if available. If our proposed guideline was used for the same group of patients included in this study, then the number of pills initially prescribed would decrease by about 80%.

## Discussion

Using patient-reported medication consumption data, we found that the median opioid use after arthroscopic contracture release of the elbow was 10 oxycodone 5-mg pill equivalents, with 75% of patients consuming 30 or fewer pill equivalents. Almost one-quarter of the patients (23%) took no opioids after surgery. Multivariate analysis revealed that preoperative opioid use, age <60 years and inflammatory arthritis were independent predictors of increased opioid usage within 90 days of the surgical procedure.

On the basis of these results, we formulated guidelines to help surgeons determine an appropriate number of opioid pills to prescribe after arthroscopic release of elbow contracture. Our guideline arbitrarily “set the bar” at satisfying 75% of patient’s home opioid requirements and recommended a different prescription size based on the presence of risk factors for increased home opioid use. One could also set the bar higher at 80% or 90%. However, we found that there was considerable number of patients above the 75th percentile that were “outliers” and, thus, setting the bar higher may result in overprescription for most of the patients. Furthermore, given that most of the patients above the 50th percentile of consumption corresponded to patients with risk factors for increased home opioid use, we arbitrarily “set the bar” lower at the 50th percentile for patients without those risk factors. While a lower quantity of opioids in the initial prescription could theoretically increase refill requests, a discrepancy between completing initial prescriptions and requesting refills was observed in the present study and in previous studies.^11^, ^12^, ^13^ Furthermore, smaller prescriptions have been associated with lower opioid consumption without affecting patient satisfaction with pain management and decreasing addiction potential.^11^^,^^12^^,^^14^^,^^15^ However, prescribers may prefer to use a higher percentile for patients without risk factors and give a higher number of pills to reduce the refill needs based on their practice location and regional regulations regarding opioid prescriptions.

The majority of studies in opioid consumption in the shoulder and elbow literature address opioid usage after shoulder procedures, primarily shoulder arthroplasty and rotator cuff repair.^16^, ^17^, ^18^, ^19^, ^20^, ^21^, ^22^ One previous study examined risk factors for chronic opioid use after elbow arthroscopy.^23^ In this study, Rojas et al. using data from a national database found that preoperative opioid filling, fibromyalgia, and psychiatric illness were associated with an increased risk of prolonged postoperative opioid use after elbow arthroscopy, whereas patients <40 years of age and chronic obstructive pulmonary disease were associated with a decreased risk. While the preoperative use of opioids was also found to be a risk factor for increased opioid use within 90 days of surgery in our study, we found that younger patients (<60 years) were more likely to consume a higher amount of opioids within 90 days of surgery than older patients (≥60 years), which contrasts with what was reported by Rojas et al. This difference may be explained by the two studies having evaluated factors for opioid consumption at two different postoperative time frames (short vs long-term). Past research has shown that factors that have been associated with chronic opioid use have not been consistently found as risk factors for increased immediate postoperative consumption.^13^^,^^14^^,^^24^^,^^25^

In the present study, there was a considerable discrepancy between the quantity of opioids prescribed versus those consumed, with a marked tendency toward overprescribing. Only one-third (34%) of the opioid pills prescribed were taken, and almost all patients (96%) had opioid medication left over at the time of the final follow-up. This is in line with previous studies showing that opioids are often prescribed in excess after surgery, with estimates indicating that between two-thirds and three-quarters of the opioid pills prescribed go unused.^11^^,^^26^, ^27^, ^28^ The large discrepancy between the quantity of opioids prescribed and that which is consumed underlines an opportunity for surgeons and the healthcare team to decrease prescription amounts and minimize the risk of opioid use disorders such as opioid dependence, abuse, or overdose.

Contrary to our hypothesis, the rehabilitation protocol did not appear to be associated with opioid consumption or pain scores within 90 days of surgery except for a difference in favor of the CPM group during the two first postoperative days. However, this difference may be explained by the fact that patients in the CPM group had a brachial plexus block during the first 48 hours, and patients in the PT group did not. Whether the brachial plexus block had an effect on opioid consumption after discharge cannot be ascertained, given that the rehabilitation protocols differed between the two groups and that may have also affected the results. In addition, the lack of differences between groups may be due to a lack of power as opioid consumption and pain scores were exploratory outcomes of the RCT, and thus, it was not powered for differences in these variables.

The findings of this study contribute to the clinical practice and to the growing evidence on opioid consumption after orthopedic procedures and provides surgeons as an objective guideline on the minimum necessary quantity of opioids that should be initially prescribed after arthroscopic contracture release of the elbow. A major strength of this study is related to the longitudinal nature of the data that was prospectively collected in the context of a RCT. However, a number of factors should be carefully considered when generalizing the results of this study. While the eligibility criteria of this RCT are representative of a broad spectrum of patients undergoing arthroscopic release of elbow contracture, exclusion criteria should be taken into consideration when generalizing the results of this study. Additionally, a number of patients underwent concomitant procedures at the time of contracture release, which may have affected the postoperative pain and the results of the study. However, the need for additional procedures is common in patients undergoing arthroscopic release of elbow contracture, and the need of additional procedures at the time of contracture release was not associated with opioid consumption in the multivariate analysis. Finally, patients should be educated as to these expectations and the important societal and medical reasons for approaching pain management in this way.

### Limitations

This study does have limitations. First, using patient-reported medication consumption data is prone to bias if patients did not accurately log their medication consumption. In an attempt to mitigate this effect and to confirm completion of their diaries, patients received a reminder call every week for the 90 days by the trial coordinator. While we did not verify the patient-reported pill counts, Wojahn et al. showed in a previous study of opioid consumption that the discrepancy between patient-reported pill counts and pill counts by research staff were minimal.^13^ Second, there is a potential impact of the Hawthorne effect^29^ in this group of patients that may result in a decreased opioid consumption, as patients were not blinded to the participation in the study and given that opioid consumption is a controversial issue currently. Third, the concomitant use of over-the-counter analgesics and the analgesic effect of indomethacin used for heterotopic ossification (HO) prophylaxis may have influenced opioid consumption. However, the dose and frequency of indomethacin were standardized in all patients, and we did not find an association between the use of over-the-counter analgesics and opioid consumption. Finally, a brachial plexus block was only used in the patients who received CPM, and thus, the effect of the block on the pattern of opioid consumption after discharge could not be determined as it may be confounded by the rehabilitation protocol. However, the finding that patients who received CPM consumed less opioids while the block was in place during the first 48 hours suggests that the use of peripheral anesthetic blocks may be a useful strategy to further decrease opioid consumption after elbow contracture release, regardless of the postoperative rehabilitation protocol.

### Conclusions

This study suggests that most patients undergoing arthroscopic release of elbow contracture use relatively few opioid pills after surgery. Use of an evidence-based guideline could decrease opioid prescriptions substantially while still effectively treating patients’ pain.

## Supplementary Data

ICMJE author disclosure forms
