# Supplementary material for: Patients Use Fewer Opioids Than Prescribed After Arthroscopic Release of Elbow Contracture: An Evidence-Based Opioid Prescribing Guideline to Reduce Excess
Source: Arthrosc Sports Med Rehabil. 2021 Nov 17;3(6):e1873–82. doi: 10.1016/j.asmr.2021.09.002 (PMC8689263; doi:10.1016/j.asmr.2021.09.002)
Supplement: Supplemental File [file mmc2.docx]

**Supplemental File**

***Complete list of exclusion criteria***

1. Contraindication to use of CPM or regional brachial plexus block, such as bleeding diathesis, use of anticoagulants or severe restriction in shoulder range of movement.
2. Progressive or recalcitrant neuropathy or neuritis
3. Pre-existing factors that might limit ability to completely participate in rehabilitation such as a neuromuscular or psychosocial condition.
4. Progressive or recurrent contracture due to inflammatory disease such as rheumatoid arthritis, juvenile idiopathic arthritis, or chondrolysis.
5. Altered anatomy that might limit elbow motion, independent of the condition being treated, such as dysplasia, malunion, osteonecrosis, and congenital deformity.
6. A reasonable restoration of motion and function could not be expected.
7. Inadequate postoperative regional anesthesia.
8. Intra-operative or postoperative complication that could affect outcome.
9. Injury or disease in the postoperative period that could affect elbow function
10. Not possible to have postoperative physical therapy appointments
11. Significant portion of the procedure performed in an open manner.
12. Women that were breastfeeding knew that they were pregnant
13. Current or prior septic arthritis

***Details of trial interventions***

- Patients in both the CPM and PT groups were placed in a bulky jones dressing with an anterior “I-beam” splint.
- Patients in the CPM group received and indwelling axillary catheter for a continuous brachial plexus block for a duration of 48 hours and were admitted to the hospital for 3 days.
  - Day 1: The CPM machine was set to reflect the passive ROM achieved in surgery with minimal (i.e < 1 kg) force. Patients were in the machine for 55 minutes out of every hour.
  - Patients were allowed to be out of the machine for 10 minutes and 15 minutes out of every hour on day 2 and day 3 respectively.
  - The patient was then discharged from the hospital with a home CPM program for 4 weeks on a standard protocol in which they come out of the CPM machine for as long of the elbow could tolerate without becoming swollen or painful until reaching a point where they were able to stay out of the machine for eight to ten hours. At that point, they would start using the machine for 3 sessions per day of 30 minutes then gradually decrease the number of sessions and minutes until they are free of the use of the machine. This could take up to 4 weeks.
- Patients in the PT group were discharged from the hospital on the same day of their surgery but were required to stay locally for 3 days if from out of town in order to be assessed and treated by members of our hand therapy department.
  - Day 1: mobilization of edema, soft tissue manual therapy and passive and active ROM exercises.
    - Home-therapy program: Active ROM 10-15 repetitions of all motions at least 5 times per day. Passive ROM, 1-minute holds at tolerated end range of motion 5 times at least five times per day.
  - Days 2 and 3: Same as Day 1 and evaluation of home-therapy program
  - Patients were fitted with a custom-made orthosis set to the patient’s maximal extension
  - Following the 3 days of therapy, patients would find a physiotherapist near their home where they were to be seen 3 times a week for 4 weeks while continuing daily home exercises in that period.
